# Exosomes derived from human menstrual blood-derived stem cells alleviate fulminant hepatic failure

**DOI:** 10.1186/s13287-016-0453-6

**Published:** 2017-01-23

**Authors:** Lu Chen, Bingyu Xiang, Xiaojun Wang, Charlie Xiang

**Affiliations:** State Key Laboratory for Diagnosis and Treatment of Infectious Diseases, and Collaborative Innovation Center for Diagnosis and Treatment of Infectious Diseases, School of Medicine, Zhejiang University, Hangzhou, 310003 China

**Keywords:** D-GalN/LPS, Exosome, Menstrual blood-derived stem cell

## Abstract

**Background:**

Human menstrual blood-derived stem cells (MenSCs) are a novel source of MSCs that provide the advantage of being easy to collect and isolate. Exosomes contain some mRNAs and adhesion molecules that can potentially impact cellular and animal physiology. This study aimed to investigate the therapeutic potential of MenSC-derived exosomes (MenSC-Ex) on AML12 cells (in vitro) and D-GalN/LPS-induced FHF mice (in vivo).

**Methods:**

Transmission electron microscopy and Western blot were used to identify MenSC-Ex. Antibody array was used to examine cytokine levels on MenSC-Ex. MenSC-Ex were treated in D-GalN/LPS-induced AML12 in vitro. Cell proliferation and apoptosis were measured. MenSC-Ex were injected into the tail veins of mice 24 h before treatment with D-GalN/LPS. Blood and liver tissues served as physiological and biochemical indexes. The number of liver mononuclear cells (MNCs) and the amount of the active apoptotic protein caspase-3 were determined to elaborate the mechanism of hepatoprotective activity.

**Results:**

Human menstrual blood-derived stem cell-derived exosomes (MenSC-Ex) are bi-lipid membrane vesicles that have a round, ball-like shape with a diameter of approximately 30–100 nm. Cytokine arrays have shown that MenSC-Ex expressed cytokines, including ICAM-1, angiopoietin-2, Axl, angiogenin, IGFBP-6, osteoprotegerin, IL-6, and IL-8. MenSC-Ex markedly improved liver function, enhanced survival rates, and inhibited liver cell apoptosis at 6 h after transplantation. MenSC-Ex migrated to sites of injury and to AML12 cells (a mouse hepatocyte cell line), respectively. Moreover, MenSC-Ex reduced the number of liver mononuclear cells (MNCs) and the amount of the active apoptotic protein caspase-3 in injured livers.

**Conclusions:**

In conclusion, our results provide preliminary evidence for the anti-apoptotic capacity of MenSC-Ex in FHF and suggest that MenSC-Ex may be an alternative therapeutic approach to treat FHF.

**Electronic supplementary material:**

The online version of this article (doi:10.1186/s13287-016-0453-6) contains supplementary material, which is available to authorized users.

## Background

Clinical fulminant hepatic failure (FHF) causes relatively high mortality and affected patients present with very severe clinical symptoms, such as coagulopathy, jaundice, and multiorgan failure. However, the only clinical treatment for FHF is liver transplantation, which is limited by a shortage of donor livers [1].

D-galactosamine (D-GalN) and lipopolysaccharide (LPS) co-induce (D-GalN/LPS) FHF in mice, producing a phenotype that copies clinical FHF. This mouse model is therefore commonly used as a test model [2]. LPS is the main component of Gram-negative bacterial cell walls, which induce very strong immunogenicity and can enhance immune response. When D-GalN/LPS are applied in mice, LPS activates immune cells in the liver, including monocytes, macrophages, and hepatic Kupffer cells [3].

Human menstrual blood-derived stem cells (MenSCs) are human menstrual blood progenitor cells (MBPCs) that are isolated from menstrual fluids [4–6]. MenSCs are similar to mesenchymal stem cells (MSCs), which have proliferative capabilities and broad multipotency, including the ability to differentiate into cell types belonging to all three germ lineages [6–8]. MenSCs exhibit higher proliferation rates than bone marrow-derived MSCs and can be easily obtained without invasive procedures [9–11]. In particular, our and other groups have reported that MenSCs induce low-level immunogenicity in clinical studies and can be expanded through at least 20 passages without genetic abnormalities [9, 12, 13]. The therapeutic potential of MenSCs has been demonstrated in several disease models, such as Duchenne muscular dystrophy, stroke, type 1 diabetes, premature ovarian failure, and myocardial infarction. These studies have suggested that MenSC-based therapies may be developed into future clinical applications [12, 14–19].

Exosomes are bi-lipid membrane vesicles that have a diameter of 50–100 nm and are secreted by various cell types. Exosomes carry a complex cargo load of proteins and RNAs that can potentially impact cellular and animal physiology. Studies have shown that exosomes are involved in complex physiological processes, such as intercellular communication, antigen presentation, and immune responses [20]. High-performance liquid chromatography (HPLC) and dynamic light scatter (DLS) analyses revealed that MSCs secrete cardioprotective microparticles with diameters ranging from 50 to 65 nm [21, 22]. Furthermore, Lai et al reported that the therapeutic efficacy of exosomes derived from human embryonic stem cell-derived MSCs were similar to exosomes derived from other fetal tissue sources (e.g., the limbs and kidneys), demonstrating that MSC-derived exosomes display general therapeutic properties [23].

Silymarin, a milk thistle of *Silybum marianum*, is the well-researched drug in the treatment of liver disease [24]. It has been found that silymarin has hepatoprotective, anti-oxidant, anti-inflammatory activities [25]. Silymarin is in our study as a reference drug to compare the beneficial effects achieved by human menstrual blood-derived stem cell-derived exosomes (MenSC-Ex).

Because of these advantages, we sought to investigate the therapeutic potential of MenSC-Ex in D-GalN/LPS-induced FHF. There were two purposes to this study. First, we sought to investigate the therapeutic effects of MenSC-Ex on AML12 cells (in vitro) and D-GalN/LPS-induced FHF mice (in vivo). Second, we sought to identify the mechanism underlying the MenSC-Ex-mediated inhibition of liver apoptosis.

## Methods

### Animals

Six- to eight-week-old male C57BL/6 mice were purchased from Sippr-BK Laboratory Animal Corporation (Shanghai, China). The mice were fed food and water ad libitum and housed under standard conditions with a 12 h light and 12 h dark cycle. All animal experiments were approved by the Laboratory Animal Center of The Tab of Animal Experimental Ethical Inspection of the First Affiliated Hospital, College of Medicine, Zhejiang University.

### Cell culture

MenSCs were isolated and maintained as previously described [4, 12]. The MenSCs were collected and cultured in Chang Medium (S-Evans Biosciences, Hangzhou, China). The MenSCs used in the experiments were at the fourth to eighth passage.

The mouse AML12 hepatocyte cell line was generously provided by the Stem Cell Bank of the Chinese Academy of Sciences. AML12 cells were cultured in DMEM/F12 (Gibco, Waltham, MA, USA) supplemented with 10% fetal bovine serum (FBS; Gibco), 100 IU/mL of penicillin (Sigma-Aldrich, St. Louis, MO, USA), 100 μg/mL of streptomycin (Sigma-Aldrich), insulin, transferrin, selenium (ITS) Liquid Media Supplement (Sigma-Aldrich, USA) and 40 ng/ml dexamethasone (Sigma-Aldrich, USA).

### Identification of MenSCs using flow cytometry

The expression of isolated MenSCs surface markers was evaluated using fluorescence-activated cell sorting (FACS). Briefly, 5 × 10^5^ cells were collected and washed twice with stain buffer (BD Biosciences, San Jose, CA, USA). MenSCs were incubated in the dark for 20 min with the following primary antibodies: PE-conjugated CD29, CD34, CD45, CD73, CD90, CD105, CD117, and HLA-DR (Becton Dickinson, Franklin Lakes, NJ, USA). The stained cells were washed twice with stain buffer, resuspended in 500 μl of stain buffer and then analyzed using a FC500 flow cytometer (Beckman Coulter, Brea, CA, USA). IgG1 (Becton Dickinson, Franklin Lakes, NJ, USA) was used as an isotype control for the anti-CD29, anti-CD34, anti-CD45, anti-CD73, anti-CD90, anti-CD105, and anti-CD117 antibodies. IgG2a (Becton Dickinson) was used as the isotype control for the anti-HLA-DR antibody. The results were analyzed using FlowJo software (Tree Star, Inc., Ashland, OR, USA).

### CFU-F assay

The colony-forming unit-fibroblast (CFU-F) assay was determined as described previously [26]. MenSCs plated at 50, 150, or 250 cells per square centimeter. After 15 days of culture, cells were stained with 20% crystal violet solution for 15 min at room temperature. After phosphate-buffered saline (PBS) wash, the numbers of individual colonies were counted. Three independent experiments were performed.

### Isolation and identification of MenSC-Ex

When MenSCs reached 70–80% confluence, the cells were cultured for an additional 24 h. The conditioned medium was collected and centrifuged at 2000 g for 20 min to remove dead cells and cell debris. The supernatant was filtered through a 0.22-μm pore filter (EMD Millipore, Billerica, MA, USA) and concentrated according to a 30 KDa molecular weight cutoff (MWCO) (EMD Millipore) by centrifugation at 4000 g for 60 min. A 1/5 volume of ExoQuick-TC Exosome Precipitation Solution (System Biosciences, Inc., Palo Alto, CA, USA) was added to the supernatant and it was incubated overnight. The mixture was centrifuged at 1500 g for 30 min, which aspirated the supernatant, and spun down at 1500 g for 5 min to remove residual ExoQuick-TC. The exosome-enriched fraction was diluted with 100 μl PBS and stored at -80 °C. The protein content of the concentrated exosomes was determined using a BCA protein assay kit (Pierce, Waltham, MA, USA). MenSC-Ex were identified using transmission electron microscopy. The MenSC-Ex were confirmed to express the exosome marker tetraspan molecules [CD63 (Abcam, Cambridge, UK) and tsg101 (Abcam)] using Western blot analysis.

### Cellular uptake and in vivo tracking of MenSC-Ex

MenSC-Ex were labeled with a 1:10 ratio of Exo-Green (System Biosciences, Inc.). The exosomes solution was incubated at 37 °C for 10 min. A 100 μl volume of ExoQuick-TC was added to stop the labeling reaction. The labeled MenSC-Ex were placed on ice (or at 4 °C) for 30 min and centrifuged for 3 min at 14,000 rpm to remove the supernatant, which contained excess label. The pellet containing the labeled MenSC-Ex was resuspended in 500 μl of PBS, and at least 100 μl of the solution of labeled MenSC-Ex was added to approximately 1 × 10^5^ AML12 cells in one well of a 6-well culture plate, which was incubated for 24 h. AML12 cells were incubated with 100 μl of PBS instead of MenSC-Ex as the control. The cellular uptake of MenSC-Ex was observed under a confocal laser microscope (Carl Zeiss, Oberkochen, Germany).

XenoLight DiR (Perkin Elmer, Waltham, MA, USA) was used to track exosomes in vivo. The solution of XenoLight DiR was diluted to 300 μM in PBS. We added the diluted XenoLight DiR solution to 10 μg of MenSC-Ex in 1 ml PBS to obtain a final concentration of 2 μM. The cells were incubated with MenSC-Ex at room temperature for 30 min. A 1/5 volume of ExoQuick-TC Exosome Precipitation Solution (System Biosciences, Inc.) was then added to the supernatant for 30 min. The solution was then centrifuged for 3 min at 14,000 rpm. The pellet, which contained the fluorescently labeled MenSC-Ex, was resuspended in 100 μl of PBS. We then systemically administered 50 μg of MenSC-Ex via the tail vein. IVIS analysis (Caliper Life Sciences, Hopkinton, MA, USA) and dissections were performed after 3 h and 6 h.

### Antibody arrays

Human Cytokine G1000 arrays (AAH-CYT-G1000; RayBiotech, Norcross, GA, USA) were used according to the manufacturer’s instructions to measure the expression levels of 120 cytokines in the MenSCs and MenSC-Ex. Positive signals were captured on glass chips using a laser scanner (GenePix 4000B Microarray Scanner; Molecular Devices, Sunnyvale, CA, USA), and the observed fluorescence intensities were normalized to the intensities of the internal positive controls. These cytokines were screened using the following integrated conditions: the MenSC group compared to the MenSC-Ex group (*p* < 0.05) for samples with fluorescence intensity values that exceeded 300 (RayBiotech). Differentially expressed proteins were arranged using hierarchical clustering and represented as a heat map. The heat map was generated using R software (http://www.r-project.org/).

### Cell proliferation and apoptosis analysis

A Cell Counting Kit-8 (CCK-8, Dojindo, Kumamoto, Japan) was used to evaluate proliferation in MenSC-Ex-treated D-GalN/LPS-induced AML12 cells. After the cells were cultured for 24 h with 44 μg/ml D-GalN and 100 ng/ml LPS, the CCK-8 reagent was added to the chamber, and the cells were incubated for an additional 3 h according to the manufacturer’s protocol. The optical densities of the solutions were read at 450 nm (OD450) and measured using a multifunctional microplate reader (SpectraMax M5, Molecular Devices).

To analyze cell apoptosis, MenSC-Ex-treated AML12 cells that were cultured for 24 h with 44 μg/ml D-GalN and 100 ng/ml LPS were collected, centrifuged, and then stained with propidium iodide and Annexin V in the dark for 30 min at room temperature using a cell apoptosis Analysis Kit (Sigma-Aldrich). Cell apoptosis was then analyzed using flow cytometry.

### The animal model and MenSC-Ex transplantations

To induce FHF in mice, C57BL/6 mice (20 ± 2 g) were intraperitoneally injected with D-GalN (800 mg/kg) (Sigma-Aldrich) and LPS (50 μg/kg) (Sigma-Aldrich). Mice injected with an equal volume of PBS alone were used as the model group for the FHF model (*n* = 10 per model group). To evaluate the therapeutic efficacy of MenSC-Ex on FHF, 1 μg/μl of MenSC-Ex in PBS or PBS alone was injected into the tail veins of mice 1 day before treatment. The animals were anesthetized using sodium pentobarbital (50 mg/kg; Solarbio Bioscience & Technology, Shanghai, China) 6 hours after treatment with D-GalN/LPS. The serum samples were centrifuged at 3000 rpm for 10 min to collect clear serum to detect the levels of alanine aminotransferase (ALT) and aspartate aminotransferase (AST). A portion of liver tissue was stored in 4% paraformaldehyde for histological and immunohistochemical analysis. The remainder of the tissue samples was washed in cold saline and preserved at -80 °C for further analysis using reverse transcription-polymerase chain reaction (RT-PCR). The survival rates of the mice were determined for the 12 h period following D-GalN/LPS challenge.

### Liver function tests and ELISA

Liver function was assessed by analyzing serum alanine aminotransferase (ALT) and aspartate aminotransferase (AST) levels. ALT and AST levels were measured using commercial kits (Nanjing Jiancheng Bioengineering Institute, Jiangsu, China) according to the manufacturer’s instructions.

The levels of interleukin-6 (IL-6), interleukin-1β (IL-1β), and tumor necrosis alpha (TNF-α) were measured in serum samples using commercially available enzyme-linked immunosorbent assays (ELISAs) (RayBiotech) according to the manufacturer’s instructions.

### Histological, immunohistochemistry and TUNEL staining

Liver tissues were harvested from mice 6 h after treatment with D-GalN/LPS or MenSC-Ex. Tissues were fixed in 10% buffered formalin, embedded in paraffin, sectioned to a 5-mm thickness, and stained with hematoxylin and eosin (H&E). The sections were imaged using an Olympus IX83 inverted microscope (Olympus, Tokyo, Japan) equipped with Olympus cellSens software (cellSens Standard 1.9).

To perform the immunohistochemistry test, peroxidase activity was blocked by incubating the sections in 3% H_2_O_2_ for 10 min. The sections were then pretreated via heat-mediated antigen retrieval with sodium citrate buffer (pH6.0). The tissue sections were blocked in 10% FBS for 20 min at room temperature and then incubated with rabbit polyclonal antibodies against mouse caspase-3 (Cell Signaling Technology, Danvers, MA, USA) overnight at 4 °C. The slides were washed three times with PBS for 5 min and subsequently incubated with secondary antibodies (Abcam). A solution of diaminobenzidine tetrahydrochloride (DAB kit; Maixin Biotech, Fujian, China) was used as the reaction substrate.

Apoptosis was detected in liver cells in paraffin-embedded sections using a fluorescence terminal deoxynucleotidyl transferase dUTP nick-end labeling (TUNEL) apoptosis assay kit (Vazyme, Nanjing, China) according to the manufacturer’s instructions. Stained sections were observed and photographed using an Olympus IX83 inverted microscope equipped with Olympus cellSens software. The number of positive cells was counted in six randomly selected fields per slide.

### DNA fragmentation analysis

Genomic DNA was extracted from liver samples according to the instructions supplied by the manufacturer of the DNA Ladder kit (Beyotime, Jiangsu, China). The DNA was then electrophoresed in a 1.5% agarose gel, which was stained with 0.1 g/ml ethidium bromide at 140 V for 20 min.

### Isolation of liver mononuclear cells

Liver mononuclear cells (MNCs) were isolated and prepared as previously described [27]. Briefly, the livers of C57BL/6 mice were passed through a 70-μm stainless steel mesh. The precipitated cells were resuspended in 40% Percoll (Sigma-Aldrich), gently lain over 70% Percoll and centrifuged at 2000 g for 20 min at room temperature. The MNCs were contained in and isolated from the interphase. Liver MNCs were stained using anti-mouse CD11b, CD3, NK1.1, and F4/80 antibodies (Biolegend, San Diego, CA, USA) and subjected to FACS analysis.

### Quantitative real-time RT-PCR

Quantitative real-time RT-PCR (qRT-PCR) was used to analyze mRNA expression levels using a CFX96 Real-time PCR Detection System (Bio-Rad, Hercules, CA, USA). These experiments were performed according to a previously described protocol [28]. Briefly, the reaction consisted of 1 μL of cDNA, 8.2 μL of RNAse-free water, 10 μL of SYBR® Fast qPCR Master Mix (Takara Bio Inc., Mountain View, CA, USA), and 0.4 μL of each gene-specific primer (10 mM). The primer sequences are shown in Table 1. The relative quantities of each PCR product were determined using the following equation: RQ = 2^-ΔΔCT^. GAPDH served as an internal control.

**Table 1:** Primers used for qRT-PCR analysis

Primer name	Sequence (5’-3’)	Species
GAPDH-F	AATGGATTTGGACGCATTGGT	Mouse
GAPDH-R	TTTGCACTGGTACGTGTTGAT	
IL-6-F	GGCGGATCGGATGTTGTGAT	Mouse
IL-6-R	GGACCCCAGACAATCGGTTG	
IL-1β-F	GTACATCAGCACCTCACAAG	Mouse
IL-1β-R	CACAGGCTCTCTTTGAACAG	
TNF-α-F	ACTCCCAGAAAAGCAAGCAA	Mouse
TNF-α-R	CGAGCAGGAATGAGAAGAGG	
Caspase-3-F	TACCGGTGGAGGCTGACT	Mouse
Caspase-3-R	GCTGCAAAGGGACTGGAT	

### Western blot analysis

After MenSCs were collected, the cells and MenSC-Ex were homogenized using cell lysis buffer (Cell Signaling Technology) containing 100× phenylmethylsulfonyl fluoride (PMSF) (Beyotime Biotechnology Inc., Shanghai, China). Twenty micrograms of total protein were obtained from MenSCs and MenSC-Ex and placed in separate lanes to be separated using electrophoresis on NuPAGE® Novex 10% Bis-Tris gels (Life Technologies, Carlsbad, CA, USA). The separated proteins were then transferred onto PVDF membranes (EMD Millipore). The membranes were blocked in 0.5% bovine serum albumin (BSA) for 1 h at room temperature and then incubated with primary antibodies at 4 °C overnight. They were subsequently incubated with HRP-conjugated secondary antibodies (goat anti-mouse or goat anti-rabbit, Bio-Rad) for 1 h at room temperature. Immunoreactive bands were visualized using enhanced enhanced chemiluminescence (ECL) reagent (Bio-Rad) with a Tanon-4500 digital image system (Tanon Science & Technology, Shanghai, China).

### Statistical analysis

All statistical analyses were performed using GraphPad Prism v5.0 (GraphPad Software Inc., San Diego, CA, USA). All data represent the means ± standard deviation. One-way analysis of variance (ANOVA) was used to determine differences between groups. *P* values < 0.05 (*) or < 0.01 (**) were considered to indicate statistical significance.

## Results

### Identification of MenSCs

**Fig. 1:**
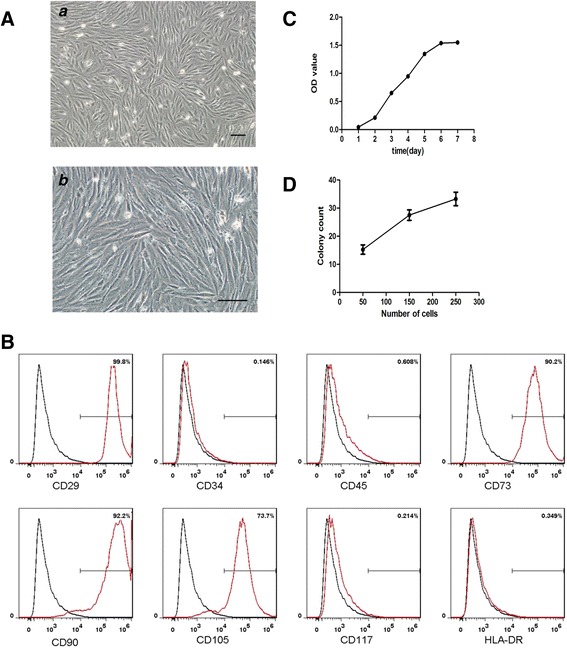
Identification of MenSCs. **A** Representative images of MenSCs shown at (*a*) scale bar = 100 μm and (*b*) scale bar = 100 μm. **B** Flow cytometry analysis of the surface markers expressed on MenSCs. **C** Growth curve of MenSCs by CCK-8 assay. **D** Colony count of MenSCs after 15 days of culture at 50, 150, and 250 cells per square centimeter

MenSCs have morphologies and immunophenotypes that are similar to MSCs. The MenSCs exhibited a spindle-shaped, fibroblast-like morphology (Fig. 1A); and they expressed high levels of CD29, CD73, CD90, and CD105 and did not express CD34, CD45, CD117, or HLA-DR (Fig. 1B). MenSCs exhibited a higher proliferation rate (Fig. 1C) and displayed enhanced colony-forming (CFU-F) ability depending on the number of cells (Fig. 1D).

### Identification of MenSC-Ex

**Fig. 2:**
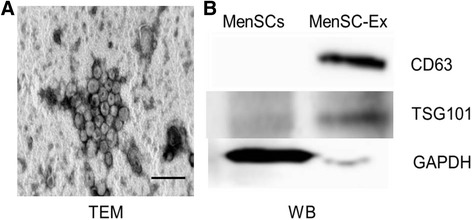
Identification of MenSC-Ex. **A** Transmission electron micrograph (TEM) of MenSC-Ex, scale bar = 200 nm. **B** CD63 and tsg101 were detected in MenSC-Ex using Western blot analysis

MenSC-Ex was prepared as previously described. Transmission electron microscopy (TEM) showed that MenSC-Ex displayed a round, ball-like shape and had diameters of approximately 30–100 nm (Fig. 2A). Western blot analysis showed that the collected MenSC-Ex expressed specific exosomal surface markers, such as CD63 and tsg101, which are not expressed on MenSCs (Fig. 2B). These results demonstrate that MenSC-Ex display specific characteristics that are identical to those described in previous studies of exosomes [29, 30].

### Differential cytokine expression in MenSCs and MenSC-Ex

**Fig. 3:**
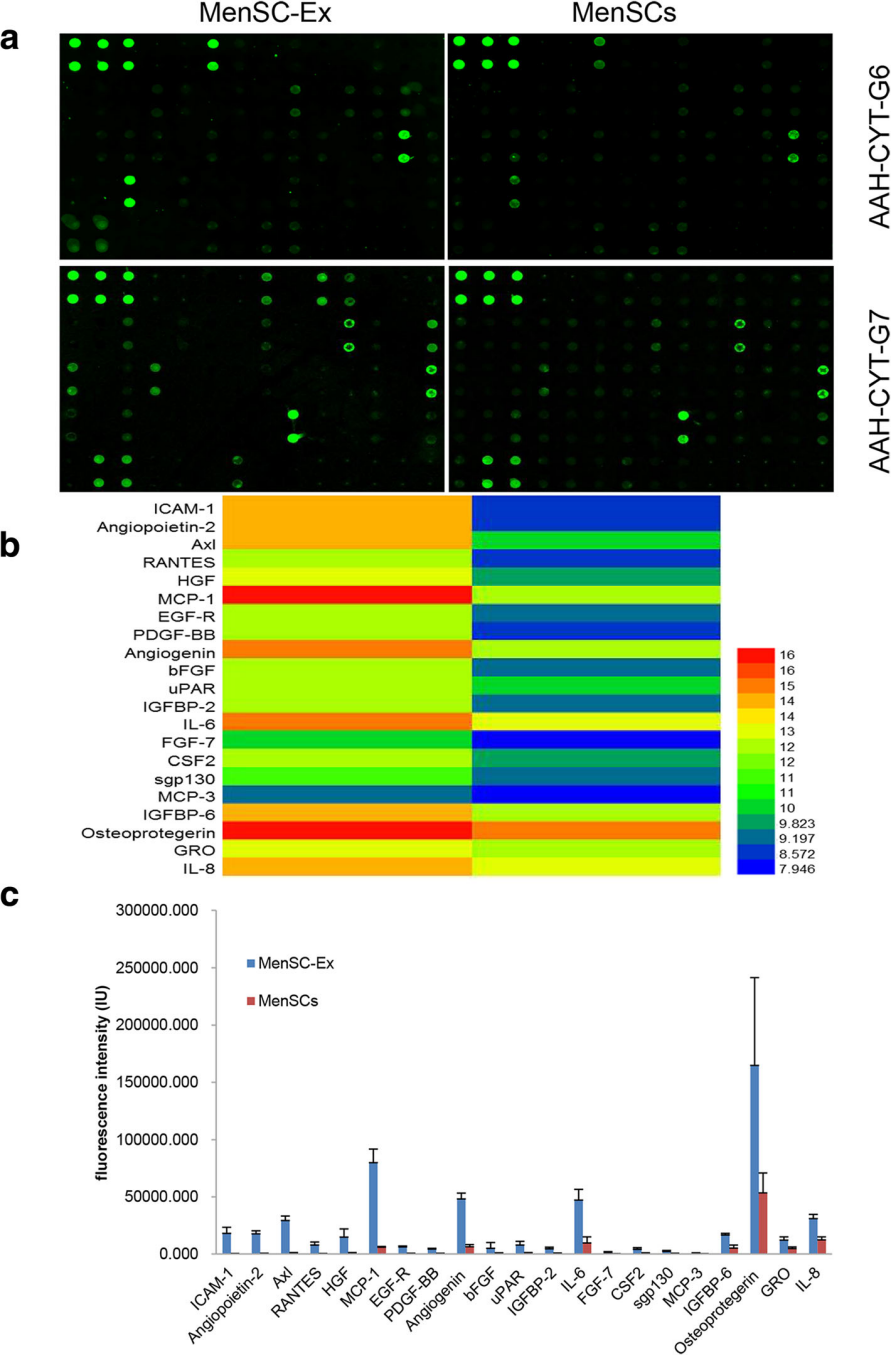
Cytokine expression in MenSC-Ex and MenSCs. **A** Representative array images are shown (*n* = 4). **B** Cytokines that were differentially expressed are shown as a heat map. **C** The fluorescence intensities of the indicated cytokines

To identify molecules expressed on MenSCs and MenSC-Ex, an antibody array was used to examine cytokine levels (Fig. 3A). Several cytokines, including intercellular cell adhesion molecule-1 (ICAM-1), angiopoietin-2, Axl, angiogenin, insulin-like growth factor-binding protein 6 (IGFBP-6), osteoprotegerin, IL-6 and IL-8, were expressed at higher levels on MenSC-Ex than on MenSCs. Among the 120 cytokines that were evaluated in this array, some were not expressed at detectable levels or were expressed at extremely low levels (data not shown). The cytokines that were differentially expressed are displayed in a heat map (Fig. 3B). The average fluorescence intensities associated with each marker are shown in Fig. 3C.

### MenSC-Ex were taken up by AML12 cells in vitro and tracked in mice in vivo

**Fig. 4:**
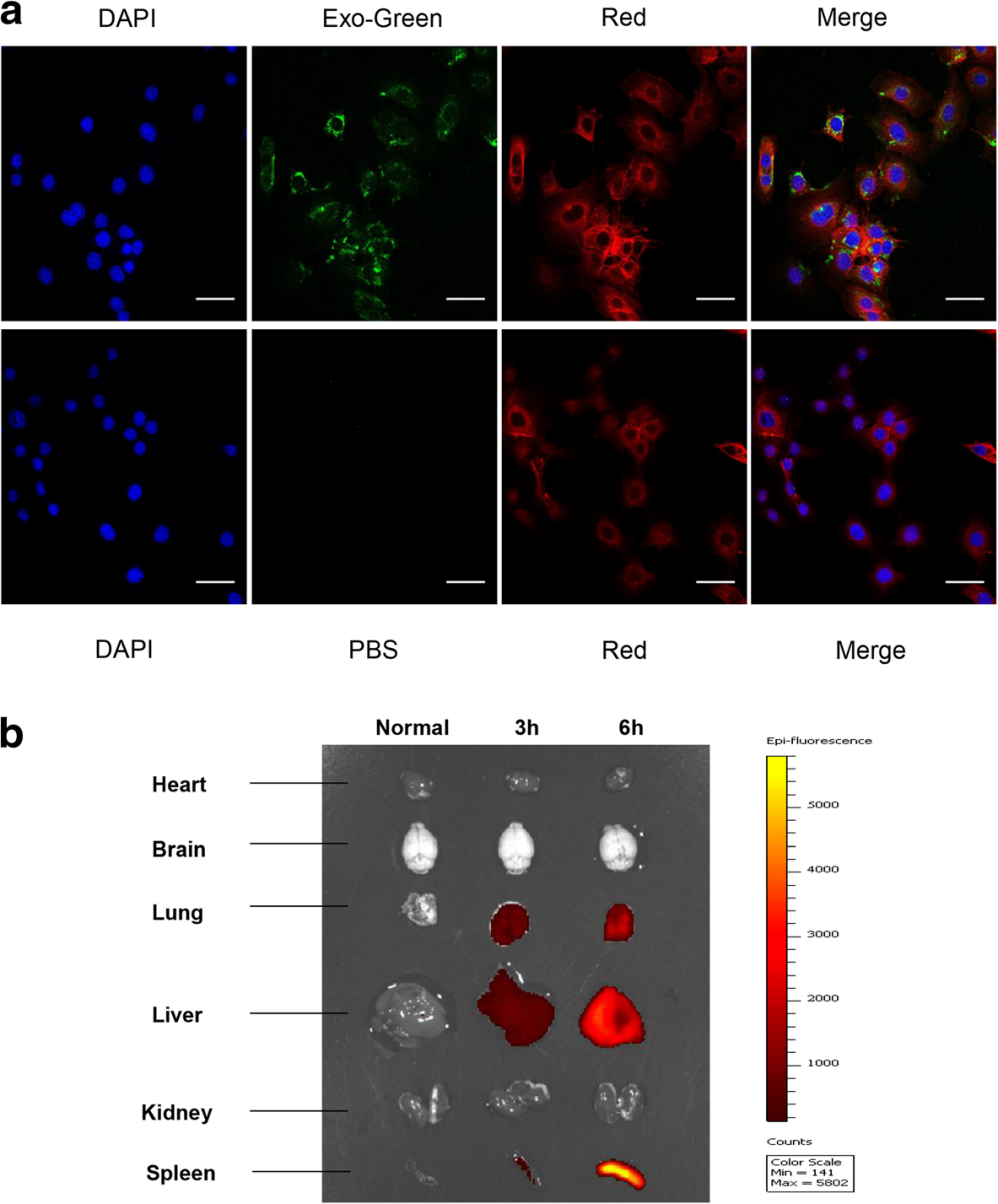
Uptake and tracking of MenSC-Ex in AML12 cells and mice. **A** Intracellular Exo-Green-labeled exosomes were detected in AML12 cells using confocal fluorescence microscopy. Scale bar = 50 μm (**B**). Analysis of XenoLight DiR-labeled exosomes after systemic administration was detected using an in vivo imaging system (IVIS)

To determine whether MenSC-Ex can be taken up by AML12 cells, we labeled MenSC-Ex with Exo-Green, a fluorescent cell linker compound that is incorporated into cellular proteins. When we incubated the Exo-Green labeled exosomes with AML12 cells, subsequently green fluorescence in the cytoplasm of almost every AML12 cell was observed (Fig. 4A). These results indicated that a significant number of exosomes were taken up by the AML12 cells in vitro.

To determine whether MenSC-Ex can be tracked in C57/BL6 mice, MenSC-Ex were labeled with XenoLight DiR. We administered the DiR-labeled MenSC-Ex into the tail veins of C57/BL6 mice, and then evaluated their distribution using in vivo imaging. At 3 h and 6 h after the injection, fluorescence was detected in the liver, the lungs, and the spleen (Fig. 4B). The in vivo fluorescence intensity indicated that the signal was retained in the liver, the lungs, and the spleen at a steady level after 3 h and 6 h. We also intravenously injected PBS as a control.

### MenSC-Ex inhibited apoptosis in D-GalN/LPS-induced AML12 cells

**Fig. 5:**
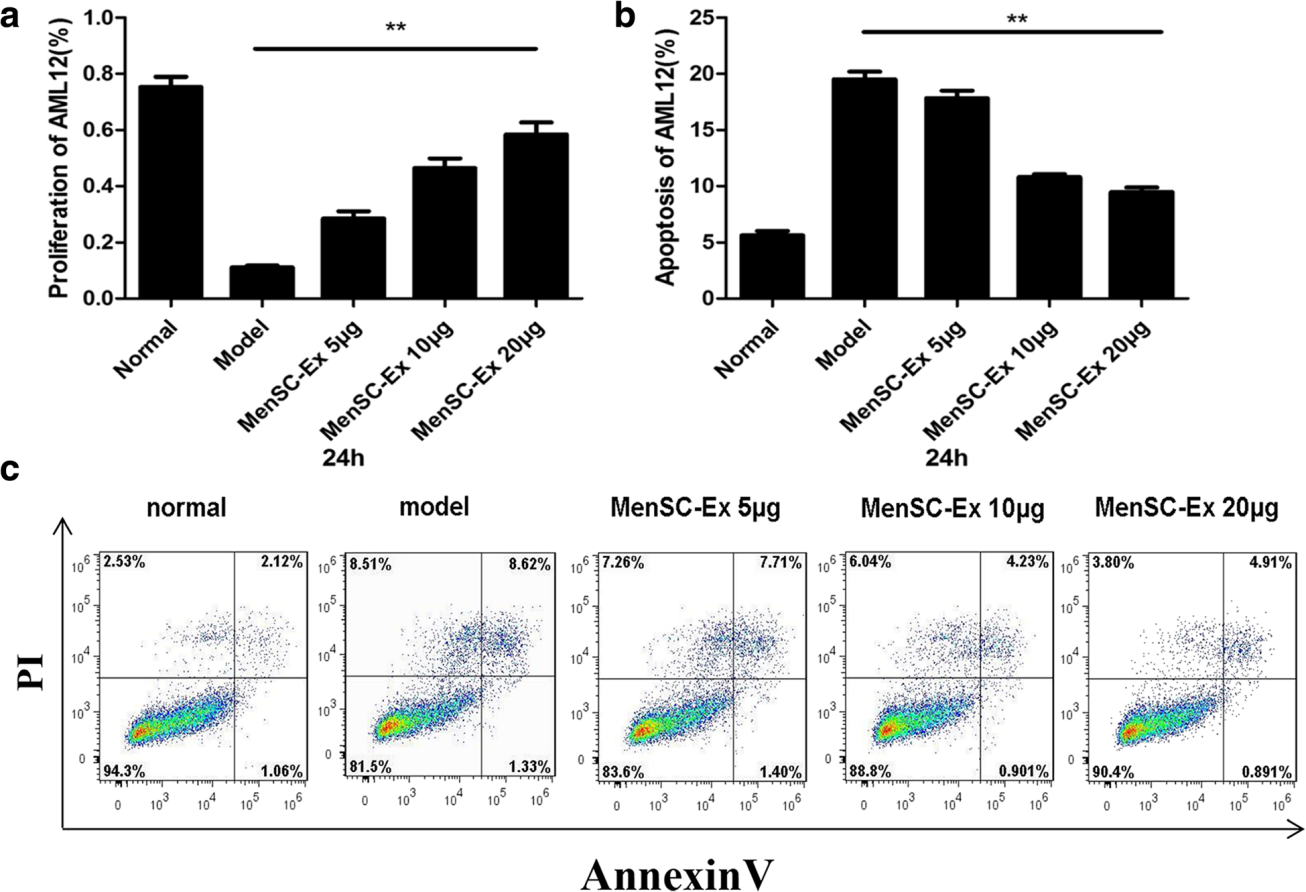
MenSC-Ex treatment inhibited apoptosis in D-GalN/LPS-induced AML12 cells. **a** The percentage of MenSC-Ex that was produced in proliferating D-GalN/LPS-induced AML12 cells. **b** Annexin V/PI staining of D-GalN/LPS-induced AML12 cells. **c** The percentage of cells undergoing apoptosis was measured. Each experiment was repeated three times; *p < 0.05

To measure the effect of D-GalN/LPS on the viability of AML12 cells, the ratio of its inhibitory effect was determined using a CCK-8 assay. Three different doses (5 μg, 10 μg, and 20 μg) of MenSC-Ex were found to inhibit the effects induced by D-GalN/LPS on AML12 cells. The data for five of the groups showed significant differences. Furthermore, the inhibitory ratio increased in a dose-dependent manner as the dose of MenSC-Ex increased. These results demonstrated that MenSC-Ex exert an anti-apoptosis effect on AML12 cells (Fig. 5A).

To determine the effects of MenSC-Ex on D-GalN/LPS-induced AML12 apoptosis, AML12 cells were pretreated with either exosomes or PBS (both in FBS-free medium) for 24 h. The cells were then co-incubated with D-GalN/LPS for an additional 6 h. Apoptosis was measured in the AML12 cells using Annexin V/PI (Fig. 5B). When cells were treated with D-GalN/LPS, the ratio of cells undergoing apoptosis in the AML12 cells was higher than in the MenSC-Ex-treated group. There were significant differences in the proportions of apoptotic cells between the three different doses of MenSC-Ex (Fig. 5C). These results suggest that MenSC-Ex inhibit D-GalN/LPS-induced apoptosis in AML12 cells.

### MenSC-Ex enhanced the survival rate and improved liver function in a D-GalN/LPS-induced mouse model of FHF

**Fig. 6:**
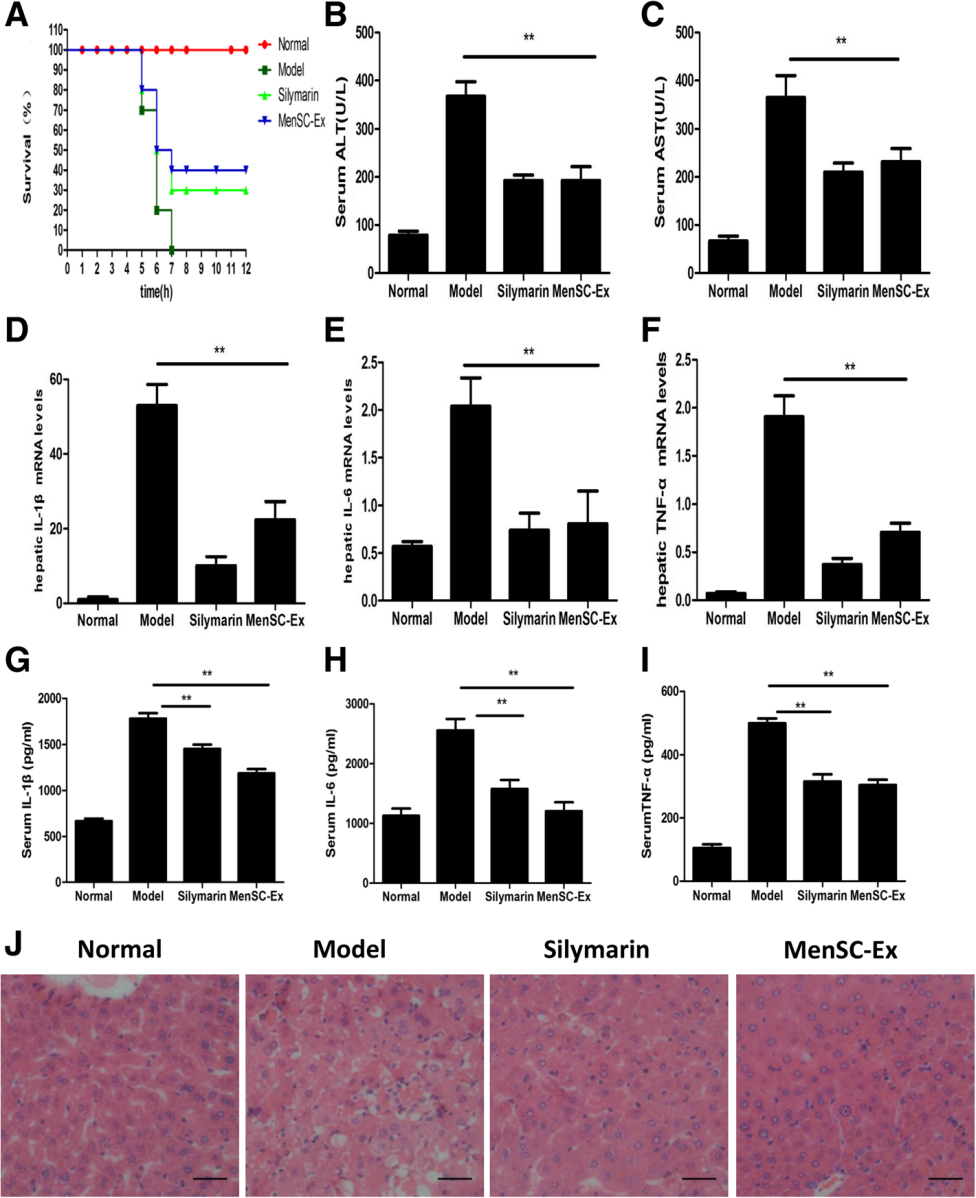
MenSC-Ex transplantation enhanced survival rates and improved liver function in a D-GalN/LPS-induced FHF mouse model. **a** Survival curves after mice were injected with D-GalN/LPS (n = 10). **b** and **c** Serum levels of ALT and AST were measured at 6 h after stimulation with D-GalN/LPS. **d**-**f** Liver levels of the IL-6, IL-1β, and TNF-α mRNAs were also detected using real-time RT-PCR at 6 h after the nice were injected with D-GalN/LPS. **g**-**i** Serum levels of IL-6, IL-1β, and TNF-α were determined using ELISA. **j** Liver sections were obtained from D-GalN/LPS-induced mice and analyzed using H&E staining (n = 10 per group, **p < 0.01). Scale bar = 50 μm

Mice began to die at 6 h after they were injected with D-GalN/LPS, and the mortality rate in these mice reached 80% within 12 h. However, mice pretreated with MenSC-Ex had significantly reduced mortality (Fig. 6A). Mice treated with silymarin, the positive control, exhibited a lower protective effect than was observed in the mice treated with MenSC-Ex.

D-GalN/LPS increased the levels of ALT and AST in the serum of C57/BL6 mice, resulting in a significant increase in liver injury over the level observed in the PBS group, the silymarin-treated group, and the MenSC-Ex-treated group. Mice pretreated with MenSC-Ex showed significantly lower levels of ALT and AST than were observed in the group treated with D-GalN/LPS alone (*p* < 0.01). There was no significant difference between the MenSC-Ex group and the silymarin group (Fig. 6B, C).

In the FHF mouse model, real-time PCR data confirmed that MenSC-Ex significantly downregulated hepatic levels of TNF-α, IL-6, and IL-1β, suggesting that MenSC-Ex prevented D-GalN/LPS-induced FHF by inhibiting the production of inflammatory cytokines (*p* < 0.01) (Fig. 6D-F). Furthermore, the results of ELISA showed that D-GalN/LPS increased the levels of the inflammatory cytokines TNF-α, IL-6, and IL-1β in C57/BL6 mouse serum. However, treatment with MenSC-Ex resulted in significantly lower levels of TNF-α, IL-6, and IL-1β than were observed in the D-GalN/LPS-induced mice (*p* < 0.01) (Fig. 6F-I).

To assess general morphological changes in the liver, liver tissue sections were mounted and stained with H&E (Fig. 6J). The normal groups showed a normal liver architecture. The groups administered with D-GalN/LPS displayed severe centrilobular focal necrosis, apoptosis and inflammation. Mice pretreated with MenSC-Ex and then induced with D-GalN/LPS showed a much lower degree of hepatocellular necrosis and inflammation than were observed in the D-GalN/LPS group.

### MenSC-Ex inhibited apoptosis in hepatocytes and the expression of caspase-3 in D-GalN/LPS-induced FHF

**Fig. 7:**
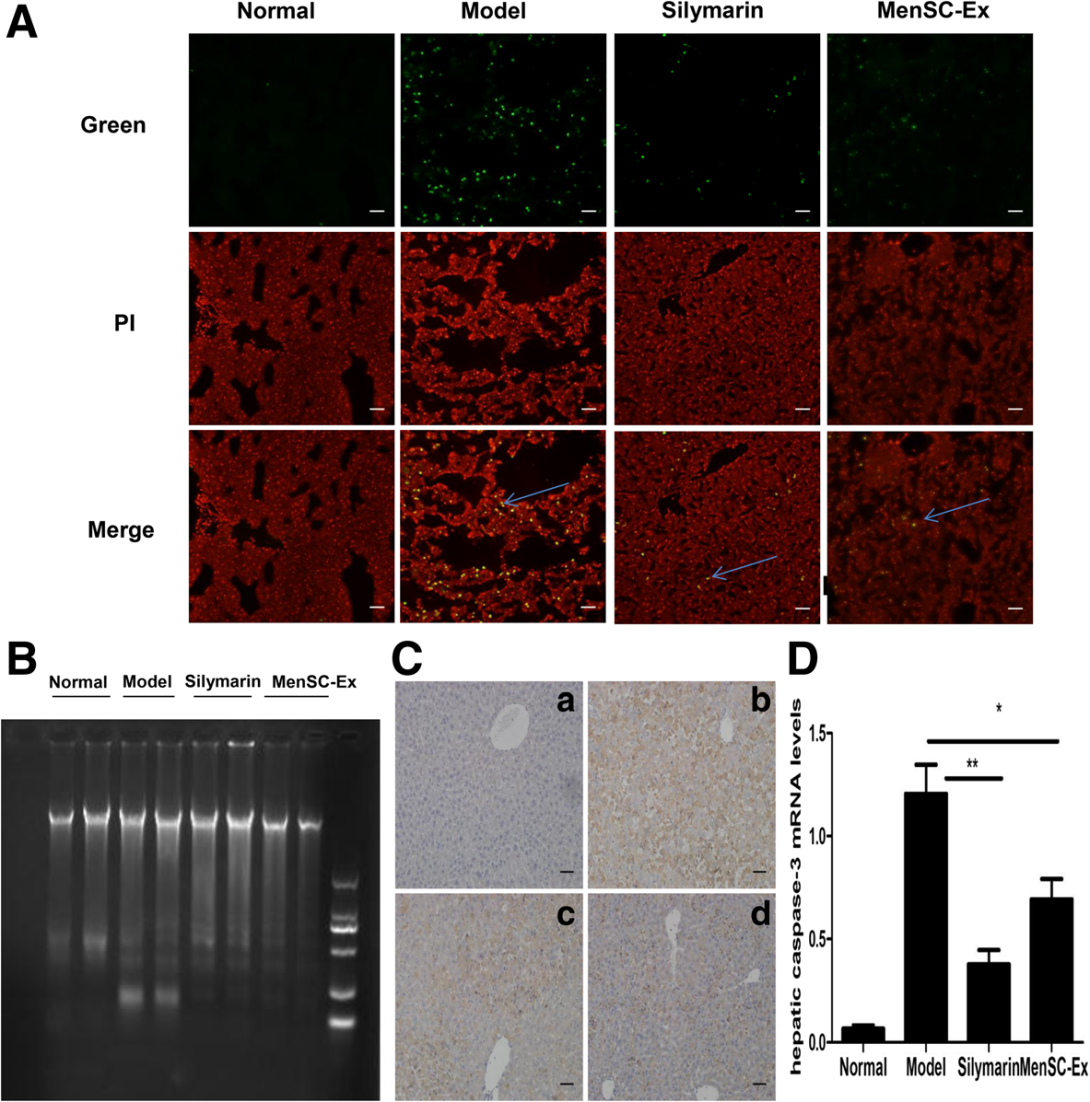
MenSC-Ex inhibited hepatocyte apoptosis and caspase-3 expression in D-GalN/LPS-induced FHF. **A** TUNEL assay showing liver sections at 6 h after D-GalN/LPS-induced FHF. Apoptotic cells were stained with *green* fluorescence, and nuclei were stained *red*, indicating PI. Scale bar = 50 μm (**B**). Analysis of genomic DNA fragmentation in liver tissues obtained at 6 h after D-GalN/LPS injection in the (*a*) normal group, (*b*) model group, (*c*) silymarin group, and (*d*) MenSC-Ex group. **C** Immunohistochemical analysis of caspase-3 expression in apoptotic liver cells at 6 h after D-GalN/LPS injection. Scale bar = 50 μm (**D**) The expression of caspase-3 was evaluated in apoptotic liver cells at 6 h after D-GalN/LPS injection (**p < 0.01)

Apoptosis was detected in hepatocytes using fluorescent TUNEL staining. A large number of TUNEL-positive hepatocytes were observed in liver tissues obtained from mice treated with D-GalN/LPS. However, few TUNEL-positive hepatocytes were observed in the livers of mice in the MenSC-Ex and silymarin groups. There were substantial differences in the number of apoptotic hepatocytes between the D-GalN/LPS and MenSC-Ex groups (Fig. 7A).

Genomic DNA fragmentation was assayed to confirm that hepatocytes were undergoing apoptosis. DNA fragmentation was observed in the livers of mice treated with D-GalN/LPS, while no DNA fragmentation was observed in the livers of the MenSC-Ex group, and only a small amount of DNA fragmentation was observed in the silymarin group (Fig. 7B).

Apoptosis is a physiological process that is involved in D-GalN/LPS-induced FHF. At 6 h after MenSC-Ex transplantation, immunohistochemistry and PCR for caspase-3 showed that there were caspase-3-positive cells in the MenSC-Ex livers (*p* < 0.01), the PBS and silymarin livers exhibited lower levels than the D-GalN/LPS group (Fig. 7C, D).

### MenSC-Ex inhibited macrophage proliferation in liver mononuclear leukocytes in D-GalN/LPS-induced FHF

**Fig. 8:**
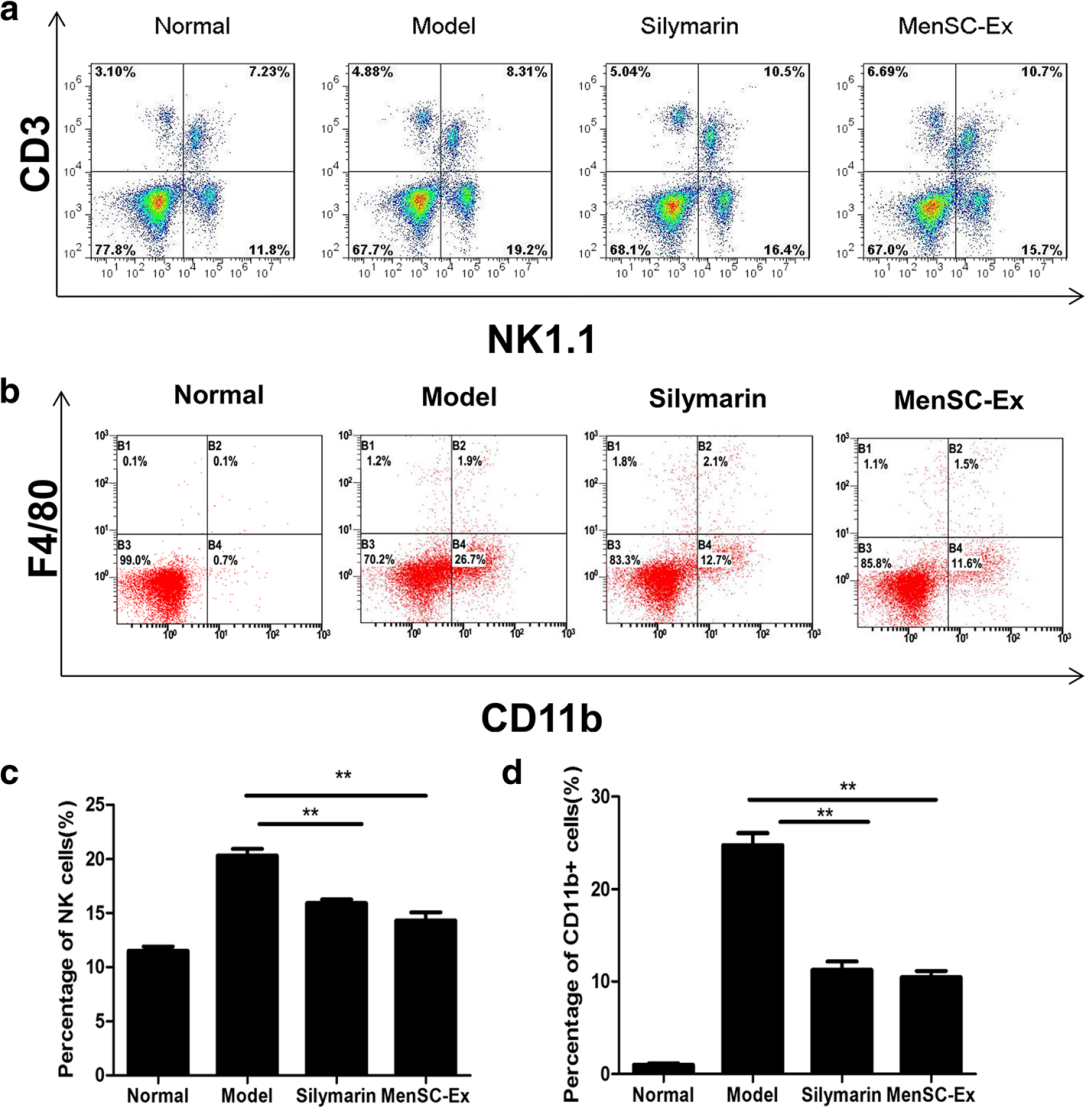
MenSC-Ex inhibited proliferation in macrophages in cultures of liver mononuclear leukocytes in D-GalN/LPS-induced FHF. **a** Mononuclear leukocytes obtained from the liver were co-stained with anti-mouse CD3 and NK1.1 antibodies at 6 h after D-GalN/LPS-induced FHF. **b** Mononuclear leukocytes in the liver were co-stained with anti-mouse CD11b and F4/80 antibodies. **c** The percentages of NK cells (CD3^−^NK1.1^+^) in the total population of mononuclear leukocytes are shown (**p < 0.01). **d** The percentages of CD11b^+^ cells out of the total population of mononuclear leukocytes are shown (**p < 0.01)

The percentage of natural killer (NK) cells that were in the liver was calculated by multiplying the percentage of CD3^−^NK1.1^+^ (NK) cells by the total number of lymphocytes per liver. NK cells (CD3^−^NK1.1^+^) and total T cells (CD3^+^NK1.1^−^) were also detected in the liver using flow cytometry. The results showed that treatment with D-GalN/LPS induced NK cells to accumulate in the liver (*p* < 0.01) (Fig. 8A, C). Administration of MenSC-Ex significantly prevented liver injury. There was no significant difference between the percentage of NK cells in the MenSC-Ex and silymarin groups. Furthermore, the severe liver injury that was triggered in the C57/BL6 mice that were treated with D-GalN/LPS indicated that NK cells could potentially mediate D-GalN/LPS-induced FHF.

Flow cytometry analysis was used to detect mononuclear leukocytes in the livers of D-GalN/LPS-induced FHF mice. CD11b is a marker of mononuclear leukocytes, and F4/80 is a specific marker of macrophages, including Kupffer cells. In normal mice, the percentage of CD11b + cells in the livers was 0.7%. After mice were stimulated with D-GalN/LPS, this percentage significantly increased to 26.7%. However, it decreased to 11.7% after the mice were treated with MenSC-Ex (*p* < 0.01) (Fig. 8B, D). There was no significant difference in the percentage of CD11b + cells between the MenSC-Ex and silymarin groups. These results demonstrated that MenSC-Ex inhibits the recruitment of inflammatory cells and reduces the number of inflammatory cells in liver. However, there were no significant differences in the numbers of CD11b+/F4/80+ cells among all four groups.

## Discussion

Because they are easy to collect, isolate, and there are no ethical considerations associated with their use, MenSCs have become a useful tool for exploring how MSCs can be used to treat tissue injuries. Our group previously reported that MenSCs are a promising therapeutic method for treating some diseases, such as liver injury and type 1 diabetes [12, 31]. Based on these benefits, we chose to use MenSCs as a source of stem-cell-derived exosomes. In this study, MenSC-Ex were used in mice, and no evidence of immune rejection was observed when MenSC-Ex were transplanted into mice. We successfully isolated exosomes from MenSCs. The MenSC-Ex had diameters of approximately 30–100 nm and expressed the CD63 and tsg101 proteins.

Several articles have shown that MSCs may perform paracrine functions that might play therapeutic roles in disease states [32–35]. It has been reported that human bone marrow MSC-derived microvesicles contain RNAs and surface proteins that had therapeutic effects in a mouse model of acute tubular injury, in which they enhanced survival [36–38]. In the present study, we used an antibody array to show that MenSC-Ex contain many cytokines, including ICAM-1, angiopoietin-2, Axl, angiogenin, IGFBP-6, osteoprotegerin, IL-6, and IL-8. Generally, IL-6 has been known to act as a pro-survival factor, which can stimulate hepatocyte proliferation [39, 40]. Osteoprotegerin could regulate bone resorption, modeling, and remodeling, which is mainly secreted by osteoblasts [41, 42]. Meanwhile, IGFBP-6 could control the differentiation of cells and prevent the apoptosis and aging of human fibroblasts [43, 44]. IL-8, a chemokine of the immune system, could recruit neutrophils, endothelial cells, and macrophages to sites of injured tissues [45, 46]. Recently, Ren et al. found that MSCs express a low level of ICAM-1, which play a critical role in MSC-mediated immunosuppression [47]. Axl is a transmembrane receptor tyrosine kinase, expressed widely in the body, which could regulate apoptosis, migration and proliferation of cells through a variety of signaling pathways [48]. According to Cai et al., angiopoietin-2 could promote the generation of new vessels in retina under hypoxia [49]. Angiogenin is capable of stimulating cell proliferation and promoting cell survival, which is predominately produced by the liver and normally circulating in human body fluid [50]. Therefore, all of the cytokines that we found were expressed in MenSC-Ex may have potentially therapeutic effects on diseases.

We evaluated liver cells in vivo and in vitro using fluorescence-labeled MenSC-Ex. Experiments in which treated cells or mice with MenSC-Ex provided initial insights into potential cellular therapies that might involve these structures. We observed clear fluorescence in AML12 cells and mice post-treatment with fluorescence-labeled MenSC-Ex. These data suggest that MenSC-derived exosomes are capable of entering into and performing functions within injured livers.

We used a D-GalN/LPS-induced mouse model of FHF to explore the mechanisms underlying clinical liver failure. In the present study, administering an injection containing MenSC-Ex prior to D-GalN/LPS reduced the circulating levels of TNF-α, IL-6, and IL-1β, decreased hepatic apoptosis, improved liver function, and eventually lowered the fatality rate in FHF mice. Our findings also demonstrated that MenSC-Ex inhibited hepatocyte apoptosis and enhanced survival in mice treated with D-GalN/LPS. Specifically, an intravenous injection of MenSC-Ex reversed organ failure. All of our results showed that MenSC-Ex functioned to affect important pharmacological characteristics.

According to the results of our experiments, the mechanisms that explain the therapeutic effect of MenSC-Ex on D-GalN/LPS-induced FHF involves the inhibitory immunomodulation of activated MNC proliferation or the activation of apoptosis-associated proteins. In the present study, one dataset suggested that there were more MNC in the D-GalN/LPS group than in the group treated with MenSC-Ex and the positive control group. One possible explanation for this result is that, in vivo MenSC-Ex may inhibit MNC proliferation, and MenSC-Ex may thereby act as crucial contributors to the observed reductions in FHF. The amount of cleaved caspase-3 expressed in the D-GalN/LPS group was lower than the amount expressed in the MenSC-Ex, but there was no difference with the positive control group. These data suggest that the activation of the apoptosis-related protein cleaved caspase-3 is another process that contributes to the mechanisms by which MenSC-Ex inhibit liver apoptosis in D-GalN/LPS-induced FHF.

NK cells are innate immune cells. A variety of artificially synthesized and natural products have been tested to evaluate their ability to recruit and activate NK cells [51]. Microbial (especially viral) invasion, tumor metastasis, and environmental materials are also capable of recruiting and activating NK cells in particular tissues or organs [52, 53]. Approximately 30–40% and 10–20% of the total number of MNC cells are NK cells in the livers of humans and mice, respectively [54, 55]. Some recent reports have shown that NK cells are involved in the pathogenesis of human hepatitis and animal models of liver injury [56–59]. D-GalN/LPS-induced FHF is a macrophage-mediated model of septic hepatitis [60, 61]. In recent years, a considerable amount of data has accumulated regarding the involvement of NK cells in the pathogenesis of sepsis, a systemic inflammatory process that is induced by bacterial lipopolysaccharide (LPS) [62]. According to data obtained by our group, D-GalN/LPS acts directly on NK cells to accelerate the increase in the number of NK cells in the injured liver. When cells or animals were treated with MenSC-Ex, the number of NK cells decreased. The reason that MenSC-Ex inhibited the increase in NK cells that was observed in the D-GalN/LPS-only treated livers remains unknown.

Caspase-3 is the most important apoptosis-associated protein [63]. The activation of caspase-3 induces an enzymatic cascade in mammalian cells that is viewed as an ‘executioner’ of apoptosis [64]. In addition, the first step in hepatocellular apoptosis requires the activation of caspase-3 [65]. In the present study, mice treated with MenSC-Ex exhibited lower levels of caspase-3 protein in the liver than were observed in the D-GalN/LPS-treated group. This finding suggests that the caspase-3-mediated death receptor pathway is one of the main mechanisms by which D-GalN/LPS induce hepatocyte apoptosis. Studies aimed at exploring the role of apoptosis proteins in D-GalN/LPS-induced liver apoptosis may provide insights into potential mechanisms through which MenSC-Ex can be used to treat D-GalN/LPS-induced FHF.

Silymarin has the therapeutic effect of restoring the normal liver functions [66]. In our study, silymarin had the ability of improving liver function in FHF mice, and its regenerative capabilities were the same as MenSC-Ex.

Although we demonstrated that MenSC-Ex ameliorated D-GalN/LPS-induced FHF in mice and explored mechanisms that may potentially underlie this effect in the present study, there are still many challenges to overcome before applications involving MenSC-Ex can be used in clinical medical practice. For example, FHF occurs rapidly, and when patients present at the hospital, they are almost always in the terminal stage of the disease. FHF causes the massive death of liver cells, and humans are much larger than mice and most other model mammals. Therefore, when MenSC-Ex are used to treat this disease, appropriate attention must be paid to the dose. In this study, MenSC-Ex reduced apoptosis in hepatocellular cells and enhanced longevity in FHF mice. However, treatments that do not renew hepatocellular tissues may be insufficient for treating FHF. Therefore, identifying methods to use MenSC-Ex in therapeutics remains a necessity for developing these cells into clinically applicable strategies.

## Conclusions

In this study, we present preliminary evidence demonstrating the potential of MenSC-Ex for reducing fulminant hepatic failure (FHF). These data suggest that MenSC-Ex transplantation deserves further study as an adjunctive or alternative approach for treating FHF.

## Additional files

Additional file 1: List of the 120 cytokines (including the G6 human cytokine antibody array and the G7 human cytokine antibody array) that were evaluated using a Human Cytokine G1000 array. *POS* positive, *NEG* negative. (TIF 4786 kb)

## Abbreviations

ALT: alanine aminotransferase
ANOVA: analysis of variance
AST: aspartate aminotransferase
BSA: bovine serum albumin
CCK-8: Cell Counting Kit-8
CFU-F: colony-forming unit-fibroblast
D-GalN: D-galactosamine
DLS: dynamic light scatter
ECL: enhanced chemiluminescence
ELISA: enzyme-linked immunosorbent assays
FACS: fluorescence-activated cell sorting
FBS: fetal bovine serum
FHF: fulminant hepatic failure
H&E: hematoxylin and eosin
HPLC: high-performance liquid chromatography
ICAM-1: intercellular cell adhesion molecule-1
IGFBP-6: insulin-like growth factor-binding protein 6
IL-6: interleukin 6
IL-8: interleukin 8
ITS: insulin, transferrin, selenium
LPS: lipopolysaccharide
MBPCs: menstrual blood progenitor cells
MenSC-Ex: human menstrual blood-derived stem cell-derived exosomes
MenSCs: human menstrual blood-derived stem cells
MNCs: liver mononuclear cells
MSC: mesenchymal stem cells
MWCO: molecular weight cutoff
NK: natural killer cells
PBS: phosphate-buffered saline
PMSF: phenylmethylsulfonyl fluoride
RT-PCR: reverse transcription-polymerase chain reaction
TEM: transmission electron microscopy
TNF-α: tumor necrosis factor-α
TUNEL: terminal deoxynucleotidyl transferase dUTP nick-end labeling
