# Supplementary figures and images for: Exosomes derived from human menstrual blood-derived stem cells alleviate fulminant hepatic failure
Source: Stem Cell Res Ther. 2017 Jan 23;8:9. doi: 10.1186/s13287-016-0453-6 (PMC5260032; doi:10.1186/s13287-016-0453-6)

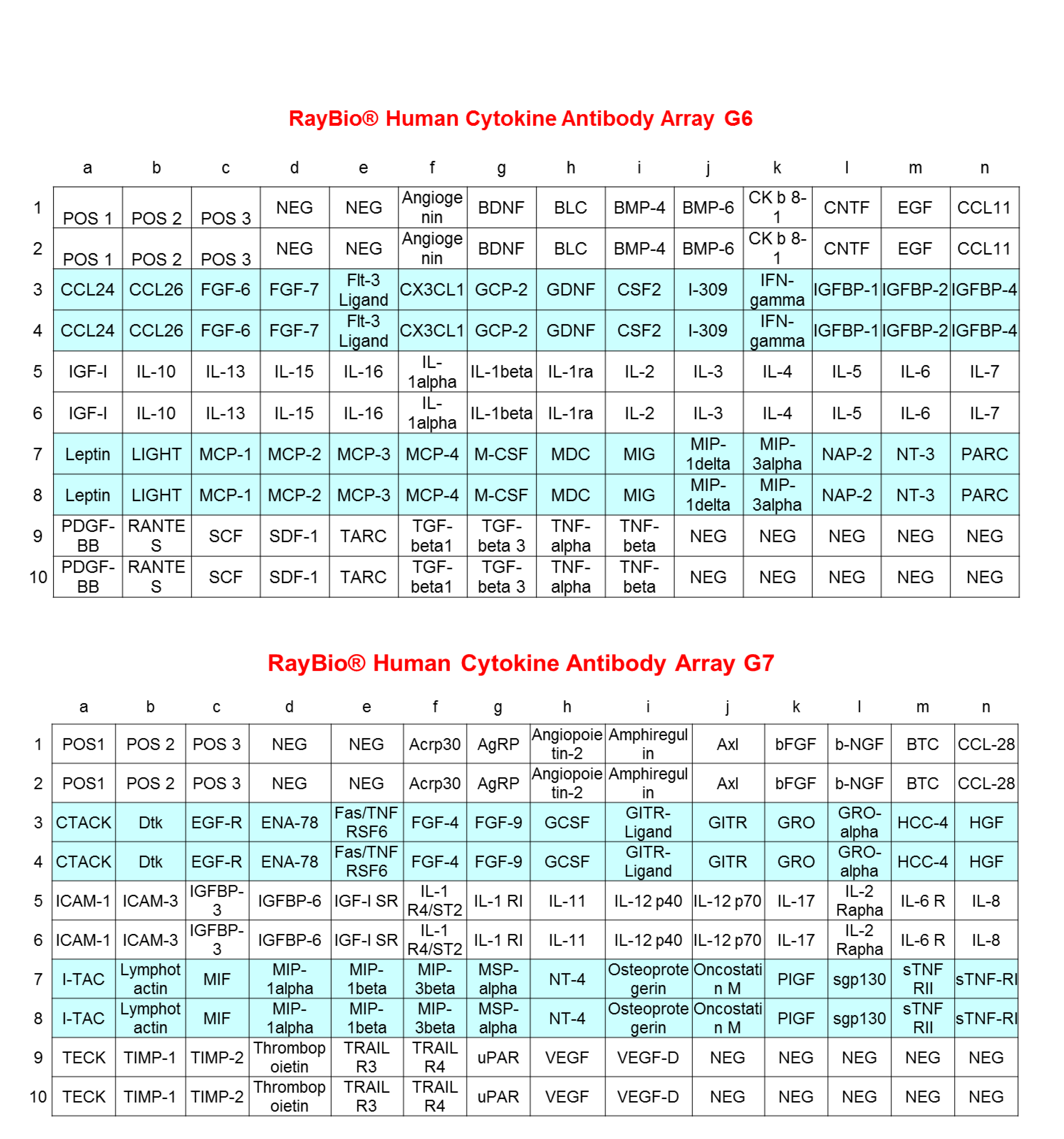

Supplement: Additional file 1: — List of the 120 cytokines (including the G6 human cytokine antibody array and the G7 human cytokine antibody array) that were evaluated using a Human Cytokine G1000 array. POS positive, NEG negative. (TIF 4786 kb) [file 13287_2016_453_MOESM1_ESM.tif]
